# CO_2_ and CH_4_ Fluxes of the Epiphytic Lichen *Bryoria simplicior* in a Permafrost-Affected Siberia: From Field Observations to Laboratory Incubations

**DOI:** 10.3390/plants15213328

**Published:** 2026-10-27

**Authors:** Oxana V. Masyagina, Svetlana Yu. Evgrafova, Natalia M. Kovaleva, Anna E. Detsura, Valery K. Kadutsky, Elizaveta V. Porfirieva, Daria O. Martynova, Anastasia I. Matvienko

**Affiliations:** 1 Sukachev Institute of Forest SB RAS, Federal Research Center “Krasnoyarsk Science Center SB RAS”, Krasnoyarsk 660036, Russiamatvienko.ai@ksc.krasn.ru (A.I.M.); 2 Melnikov Permafrost Institute, SB RAS, Yakutsk 677010, Russia

**Keywords:** epiphytic lichens, greenhouse gas, permafrost, photoperiod, *Bryoria simplicior*, thallus water content, stable isotopes, aerobic methane production

## Abstract

High-latitude climate change studies have mainly disregarded the role of epiphytic lichens in the greenhouse gas (GHG) budgets of Siberian permafrost ecosystems. In the permafrost-affected Middle Siberian region, the actual and potential fluxes of CO_2_ and CH_4_ from transplants of *Bryoria simplicior* inhabiting larch trees have been evaluated. Seasonal field measurements (June–August 2025) were combined with laboratory aerobic incubations under simulated polar-day conditions at 50%, 100%, and 200% thallus water contents. Field studies demonstrated mixed CO_2_ flux dynamics, with net CH_4_ consumption prevailing, although CH_4_ release occurred during prolonged rainfall. Incubations demonstrated potential CO_2_ and CH_4_ emissions. While *Bryoria* showed net CH_4_ uptake under dry field conditions, incubation experiments revealed its inherent capacity for both GHG release and uptake. Lichen water content and the combined light-temperature regime (photoperiod) had a significant impact on CO_2_ fluxes, whereas CH_4_ fluxes showed primarily a light-associated response. These findings highlight the susceptibility of lichen-mediated GHG exchange to moisture and light-temperature conditions, with potential implications for boreal C budgets in permafrost ecosystems under varying precipitation patterns.

## 1. Introduction

Epiphytic lichens (ELs) are ubiquitous and ecologically important components of forest ecosystems worldwide. Lichens are hypothesized to have emerged in early terrestrial life as a biofilm-like association of fungi, cyanobacteria, and other microorganisms [1]. Establishing a symbiosis between a fungal mycobiont and a photosynthetic photobiont, they inhabit tree stems and branches, enhancing biodiversity, nutrient cycling, and microhabitat formation [2,3], and are usually regarded as a reciprocal partnership between a photobiont (cyanobacteria or green algae) and a mycobiont [4]. Fungal mycobionts belong mostly to the phylum Ascomycota, and only 0.3% of lichenized fungi are known to have originated from Basidiomycota [5,6]. 85% of lichen-forming algae belong to the green algae (ca. 120 species), 10% to cyanobacteria, and the rest are brown or yellow-green algae, which are also known as photobionts in lichens [7,8,9]. The photobiont, which accounts for only approximately 10% of the thallus biomass, is critical to the survival of the entire association because it provides photosynthetic products to the mycobiont cells (in green algae) or both photosynthetic and nitrogen fixation products (in cyanobacteria) [10,11]. Lichens also harbor diverse bacterial and fungal communities whose ecological functions remain underexplored in many lichen species, especially those inhabiting high-latitude permafrost ecosystems [4,12].

Epiphytic lichens of the genus *Bryoria* (*Parmeliaceae*) are widespread and abundant in vast permafrost-affected forests of Siberia, such as those in Middle Siberia. The biomass of *Bryoria* species can range from 120 to 800 kg per hectare in boreal forests and up to 1116 kg per hectare in subalpine forests [13]. These ecosystems are experiencing some of the most rapid climatic changes among global biomes, including altered precipitation patterns and rising temperatures, while also representing a critical and vulnerable carbon (C) reservoir [14,15,16]. Lichens are well-adapted to cold climates, seasonal desiccation, and low nutrient availability [13,17]. Due to their poikilohydric nature, their metabolic processes—such as respiration and photosynthesis—are closely connected to their hydration level, which is controlled by ambient climate-related factors, such as air humidity, fog, and precipitation rather than temperature. This physiological trait makes them highly sensitive and potentially important players in the GHG balance of the ecosystems they inhabit. Beyond lichens’ well-recognized roles as bioindicators of air quality and climate change [18,19] and as important forage for reindeer, recent research has highlighted their potential involvement in biogeochemical cycles [20]. While lichens are known to respire CO_2_ and, in some cases, exhibit CH_4_ uptake or emission, the environmental drivers (temperature, moisture, light) and underlying biological mechanisms are still debated [20,21,22,23,24]. According to a recent bibliometric meta-analysis, empirical data on their seasonal greenhouse gas (GHG, e.g., CO_2_ and CH_4_) exchange dynamics under the extreme continental climate and unique photoperiod conditions (e.g., polar day) of the permafrost zone remain scarce [25]. Most existing studies have focused on lichen GHG fluxes in temperate or non-permafrost boreal regions. For example, lichen species such as *Evernia mesomorpha* have been shown to emit CO_2_ and CH_4_ in the non-permafrost Siberian region [24]. It remains unclear whether such findings can be directly extrapolated to the harsher environmental conditions of the permafrost zone, where lower temperatures, different forest composition (e.g., larch dominance), and unique light regimes may fundamentally alter the metabolic processes of the lichens and their cohabitants (microbes, fungi, yeasts, etc.). Moreover, the potential influence of microhabitat on lichen activity is often overlooked. The exposure of the tree stem (north-, south-, east-, or west-facing) to which the lichen is attached can create distinct microclimates through variations in light intensity, temperature, and moisture retention. This may potentially modulate the physiological response of lichens to larger-scale climatic drivers. To accurately model ELs’ contribution to the net GHG budget, which has implications for estimating forest ecosystem responses to ongoing climate change in high-latitude locations, the interaction of these microhabitat variables, lichen thallus water content, and photoperiod should be considered.

Methane production in aerobic environments, including from plants, fungi, and lichens, has challenged the long-held paradigm that CH_4_ is formed exclusively by methanogenic archaea under anoxic conditions [20,26,27,28,29,30,31,32,33,34,35]. Studies have indicated that research on tree stems has predominantly assessed net flux and that epiphytes, especially lichens, may be significant yet underexplored contributors to net processes [27]. Recent studies have demonstrated light-dependent CH_4_ emission from cryptogamic covers, including lichens, bryophytes, and cyanobacteria, suggesting links to photosynthetic activity and reactive oxygen species (ROS)-driven abiotic processes [36,37]. Yet the exact biochemical pathways and their sensitivity to environmental factors such as thallus water content and photoperiod remain largely unresolved. Specifically, it is unknown whether CH_4_ formation in lichens is primarily biotic (e.g., via methanogens in anaerobic microsites) [38], abiotic (e.g., via ROS-mediated demethylation) [39], or a combination thereof. However, recent ultrastructural studies have indicated that the algal and medullary layers retain a gas phase even if the thallus is fully hydrated [2]; this suggests that CH_4_ generation in lichens may occur under aerobic conditions and does not require the presence of anaerobic microsites.

We tested the following hypotheses: (*i*) *Bryoria simplicior* (Vain.) Brodo et D. Hawksw., as a dominant EL species in the larch forests (*Larix gmelinii* (Rupr.) Rupr.) of the permafrost-affected Middle Siberian region, contributes significantly to GHG exchange; (*ii*) precipitation, rather than temperature, is the primary control over in situ CO_2_ and CH_4_ fluxes via thallus hydration. To test these hypotheses, we employed an integrative approach, combining in situ measurements of CH_4_ and CO_2_ fluxes from the surface of epiphytic lichens inhabiting larch trees growing on permafrost and incubation experiments during the specific polar-day photoperiod.

Therefore, our research had the following primary objectives: (1) to quantify the in situ seasonal dynamics of GHG fluxes from *Bryoria* transplants in their natural habitat; and (2) to determine, under controlled laboratory conditions, the potential range of CO_2_ and CH_4_ fluxes in response to simulated polar-day photoperiod, varying thallus water content, and different stem exposures where the lichens were attached. By comparing our findings with data from non-permafrost regions, we aim to elucidate the key environmental controls on lichen-mediated GHG exchange in this critically important and rapidly changing environment. New data on GHG fluxes associated with EL are expected to help refine overall estimates of GHG emissions from ecosystems, leading to a better understanding of current climate processes and improved accuracy of future projections.

## 2. Results

### 2.1. Bryoria GHG Fluxes in Field Conditions

**Figure 1:**
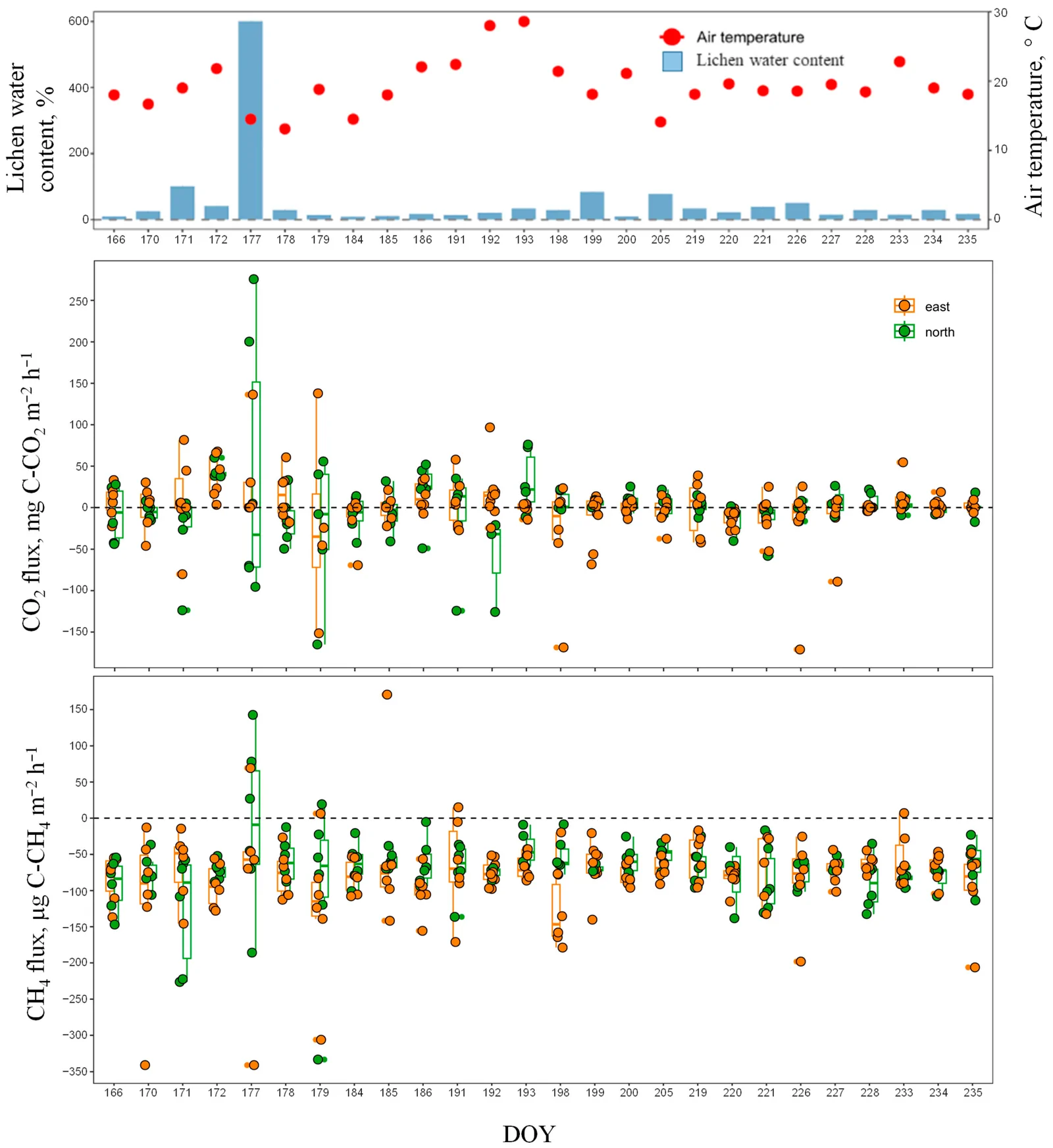
Seasonal patterns of air temperature, lichen water content, and GHG fluxes from the surface of the epiphytic lichen *Bryoria simplicior* inhabiting the bark of larch trees (DOY 166 at site D) and affixed to transplants at the Tura Experimental Station (DOY 170-235). The horizontal line inside the boxplot represents the median, the boxplot boundaries represent the 25th and 75th percentiles, and the whiskers represent the maximum and minimum values; east, eastern exposure of the lichen transplant on the stem; north, northern exposure of the lichen transplant on the stem. Air temperature and lichen water content were measured simultaneously with GHG sampling.

**Figure 2:**
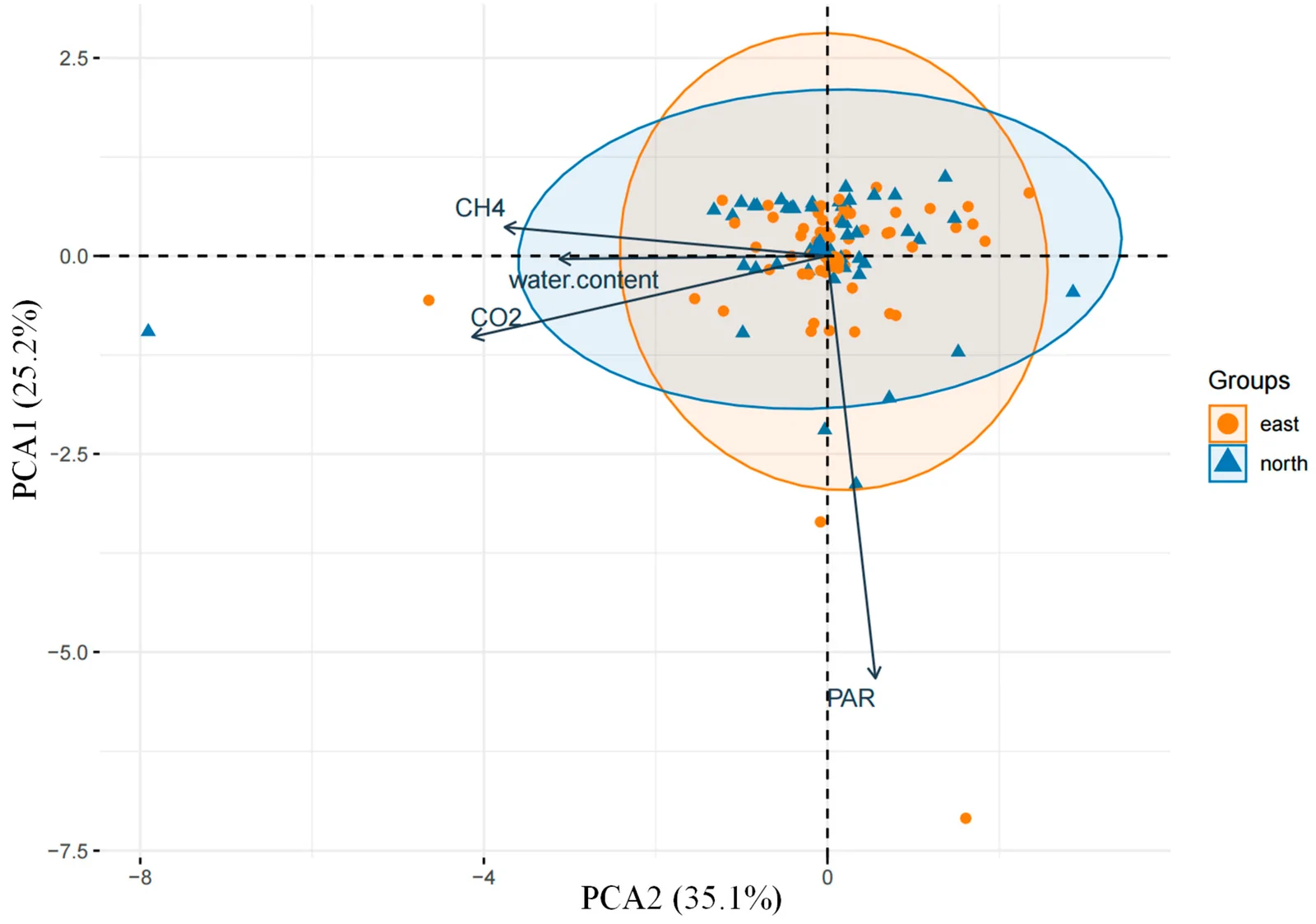
PCA analysis of the relationships between GHG fluxes by *Bryoria simplicior* lichen thalli (transplants) located on larch stem areas of different exposures, thallus water content, and PAR levels. The analysis included the initial measurements of GHG fluxes (DOY166) at site D and measurements of GHG fluxes from the surface of lichen transplants. east, eastern exposure of the lichen transplant on the stem; north, northern exposure of the lichen transplant on the stem.

**Figure 3:**
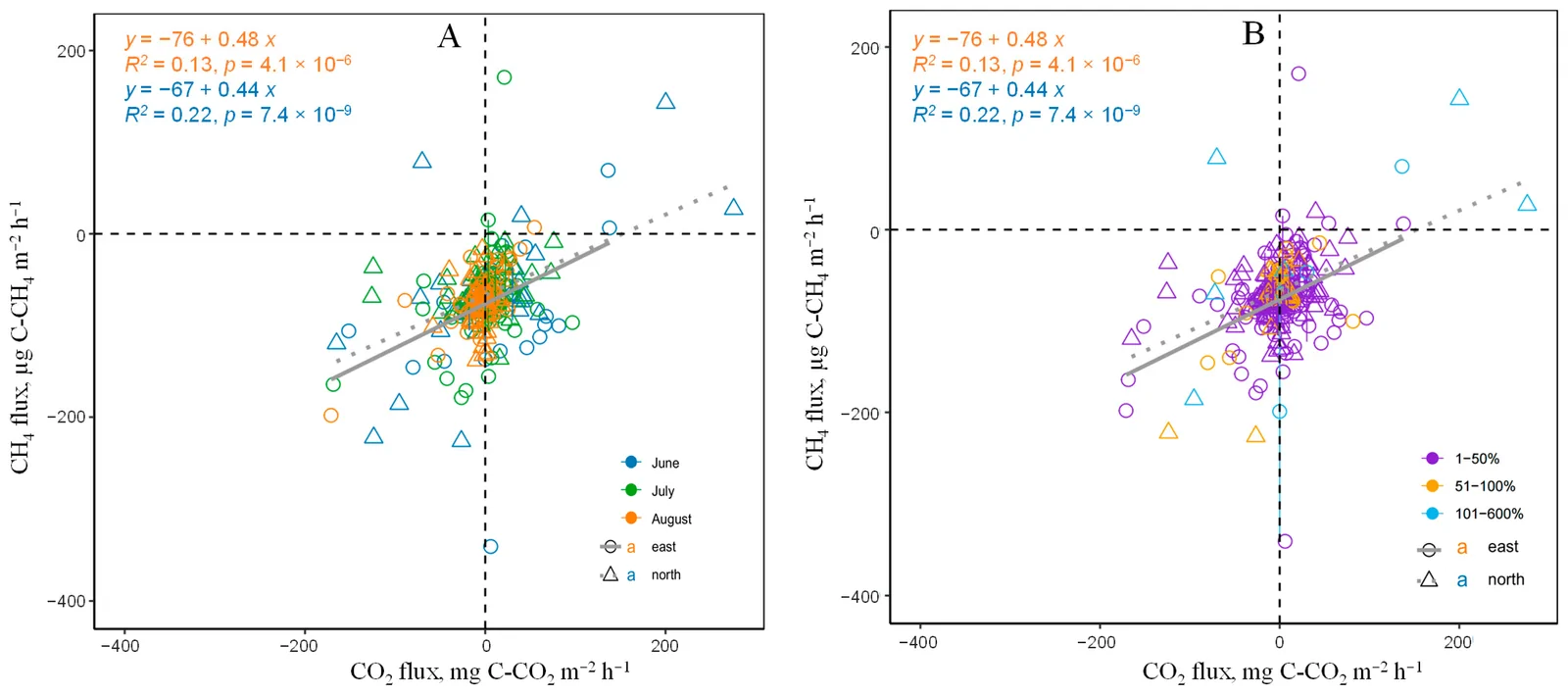
Interaction of CH_4_ and CO_2_ fluxes from the surface of *Bryoria* transplants during the growing season (**A**) and depending on lichen water content (**B**). east, eastern exposure of the lichen transplant on the stem; north, northern exposure of the lichen transplant on the stem.

Here we present two complementary datasets: (i) a single natural in situ GHG flux measurement at site D on DOY 166 and (ii) a seasonal series of transplant-based GHG flux measurements at the Tura Experimental Station from DOY 170 to 235 (Figure 1). Seasonal dynamics of GHG fluxes from the surface of lichen transplants (using *Bryoria simplicior* as an example) placed on model trees in the Tura Experimental Station (Tura settlement, Middle Siberia) showed that gas exchange of epiphytic lichen has low variability during the growing season (on average 0.4 mg C-CO_2_ m^−2^ h^−1^ per season). A key driver of short-term variability in CO_2_ fluxes is thallus water content (r = 0.17, t = 2.9454, df = 305, *p*-value = 0.003474), which varied from 8% to 600% and increased sharply after heavy precipitation. Air temperature varied from 12.9 °C to 28.6 °C within the measurement period and did not affect the CO_2_ flux dynamics during a season (r = 0.05, t = 0.85072, df = 305, *p*-value = 0.3956; Figure 1). This species consistently consumes CH_4_ during the season, with an average of −75 μg C-CH_4_ m^−2^ h^−1^, and was not altered by either thallus water content (r = 0.1, t = 1.8923, df = 308, *p*-value = 0.05939) or air temperature (r = −0.02, t = −0.31542, df = 308, *p*-value = 0.7527). PCA revealed no effect of stem exposure on *Bryoria* GHG fluxes over the season (Figure 2). Water content positively influenced GHG fluxes, and PAR did not (for CO_2_: r = 0.03, t = 0.49412, df = 305, *p*-value = 0.6216; and for CH_4_: r = −0.02, t = −0.28028, df = 308, *p*-value = 0.7795; Figure 1 and Figure 2). The minimal variation of the GHG fluxes was observed at the end of the growing season (August, Figure 3A), and maximal variation in GHG fluxes was attributed to the beginning of the growing season (June−July). The high lichen water content of transplants was associated with strong emissive GHG fluxes. The majority of seasonal GHG fluxes from the surface of the transplants were low uptake fluxes connected with lichen water content values below 100% (Figure 3B).

### 2.2. Bryoria GHG Fluxes in the Lab Incubations

**Figure 4:**
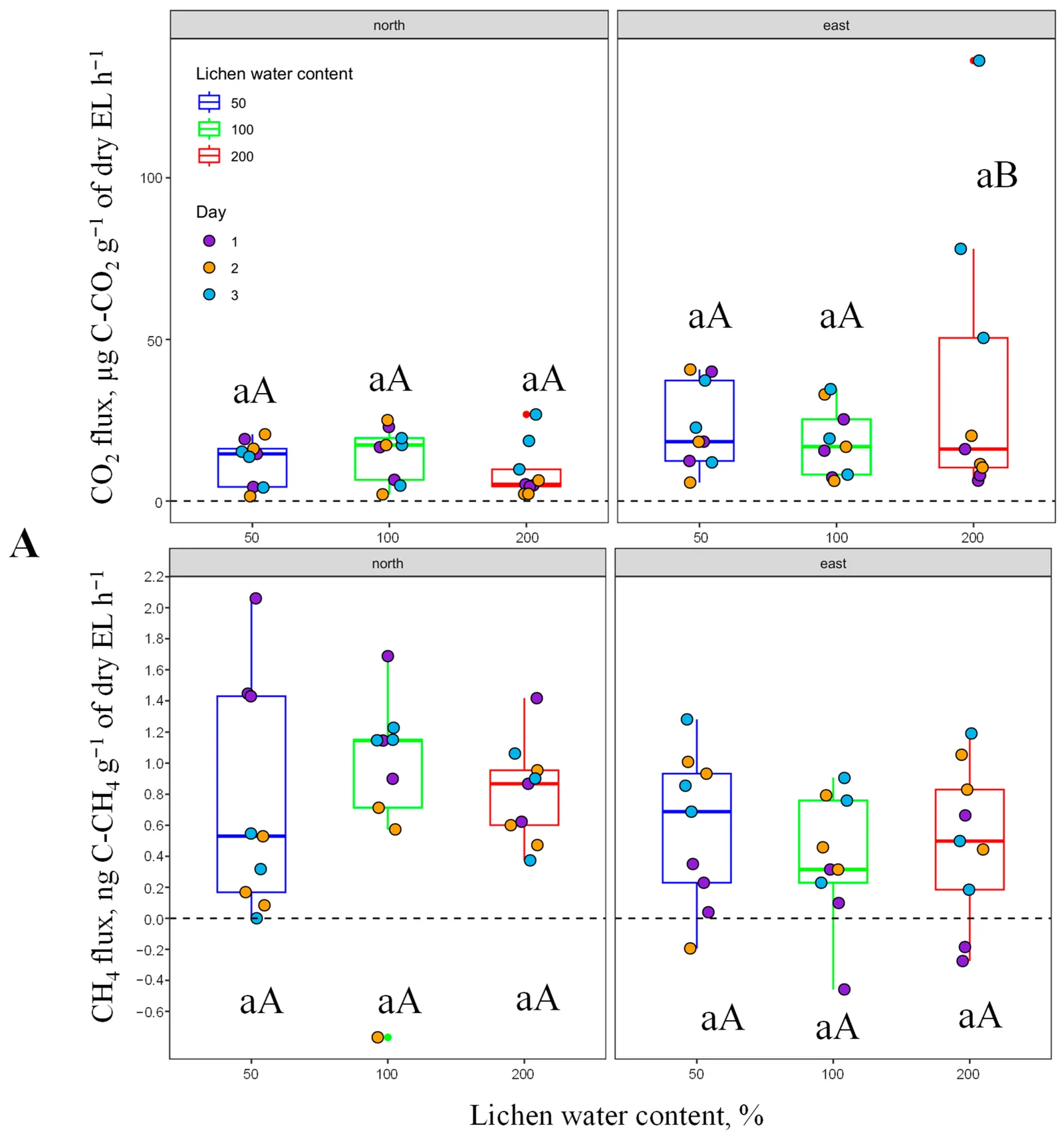

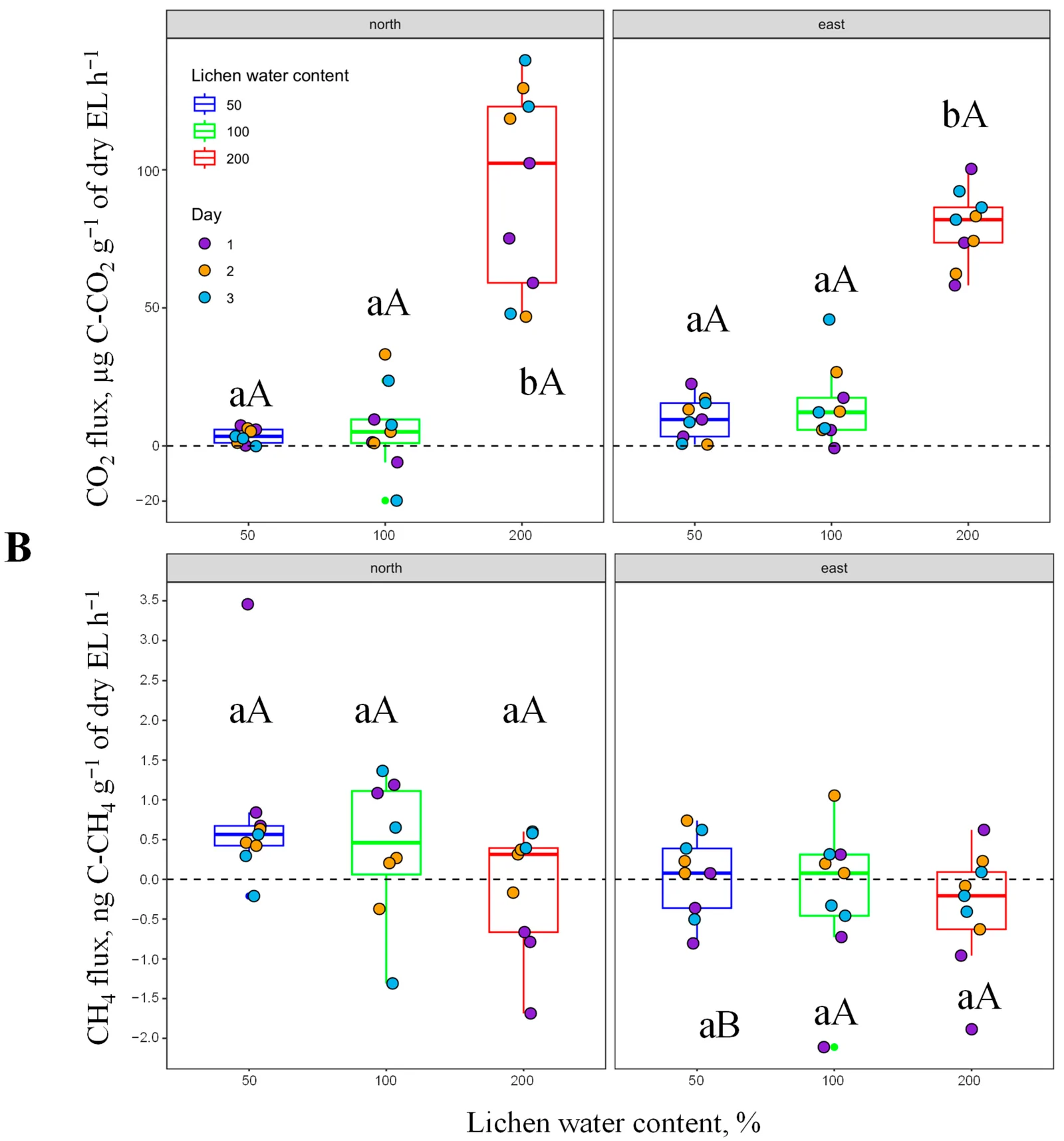
Boxplots of CO_2_ and CH_4_ fluxes from the surface of the epiphytic lichen *Bryoria simplicior* collected from larch stem areas of varying exposure (northern or eastern) and incubated under controlled photoperiod conditions. (**A**)—after 2-h exposure under illumination (100 μmol photons m^−2^ s^−1^); (**B**)—after 2-h twilight exposure (10 μmol photons m^−2^ s^−1^). east, eastern exposure of the lichen transplant on the stem; north, northern exposure of the lichen transplant on the stem. The horizontal line inside the boxplot represents the median, the boxplot boundaries represent the 25th and 75th percentiles, the whiskers represent the maximum and minimum values, and small colored dots represent outliers. Small letters indicate differences in GHG fluxes among lichens of different thallus moisture content but the same stem exposure (northern or eastern) according to the Wilcoxon paired test. Capital letters describe differences in GHG fluxes among lichens exposed to different stem exposures with the same thallus moisture content according to the Wilcoxon paired test.

The impact of stem exposure on CO_2_ flux from the surface of *Bryoria* was demonstrated by incubation experiments that imitated the polar day. Under illumination with a thallus water content of 200%, CO_2_ flux was considerably higher in lichens of eastern exposure compared to north-exposed lichens (Figure 4A). Yet, we observed no CO_2_ photoassimilation under the illumination phase. Simultaneously, incubation under twilight conditions showed CO_2_ uptake by lichens of 50–100% thallus water content (Figure 4B). CO_2_ flux rates ranged from −19 to 140 μg C-CO_2_ g^−1^ h^−1^.

Methane emissions were observed throughout the experiment with maximum values of up to 3.5 ng C-CH_4_ g^−1^ h^−1^. It was significantly higher after 2-h daylight exposure (Figure 4A) than under the twilight conditions (Figure 4B). CH_4_ fluxes (–2.1 to 3.5 ng C-CH_4_ g^−1^ h^−1^; Figure 4A,B) were generally independent of thallus moisture content and stem exposure, except for the lichens of 50% thallus moisture content, where the north-exposed items produced more CH_4_ than those exposed to the east. Empty vials without lichens served as a control to monitor background gas exchange. Background fluxes were consistently negligible (<5% of sample fluxes) and did not differ systematically between light and twilight phases, confirming that fluxes were lichen-derived.

**Figure 5:**
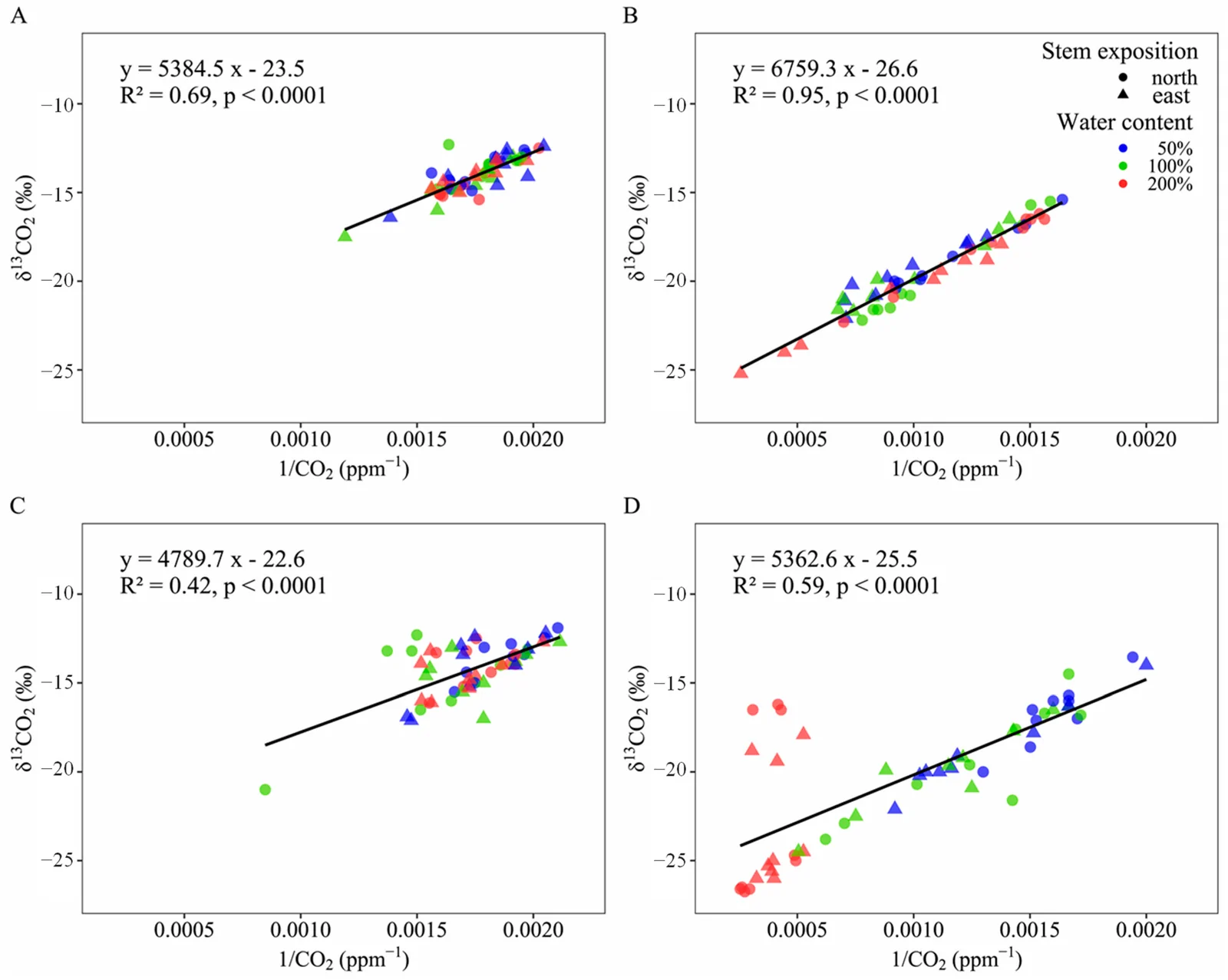
Keeling plots of δ^13^CO_2_ in the headspace of a vial containing epiphytic lichens incubated under daylight (panels (**A**,**B**)) and twilight conditions (panels (**C**,**D**)). Data are presented before (left column: (**A**,**C**)) and after the 2-h incubation (right column: (**B**,**D**)). Dots are colored by thallus water content (50% (blue), 100% (green), and 200% (red) of dry weight) and have varying shapes based on stem exposure (north, circle; east, triangle), where the lichen was adhered. Solid black lines are ordinary least squares (OLS) linear regression fits of δ^13^C to the inverse gas concentration (1/CO_2_). Regression equations and statistical parameters (R^2^, p) are shown.

Under both illumination and twilight conditions, Keeling plot analysis revealed significant linear relationships between δ^13^CO_2_ and 1/CO_2_ before and after 2-h incubation (illumination: R^2^ = 0.69–0.95, *p* < 0.0001; twilight: R^2^ = 0.42–0.59, *p* < 0.0001) (Figure 5A–D). CO_2_ concentration increased from 575 ± 60 ppm to 1061 ± 515 ppm under illumination and from 589 ± 103 ppm to 1475 ± 1024 ppm under twilight, reflecting predominantly respiration. The mean δ^13^CO_2_ values shifted towards lighter signatures from −14.1 ± 1.1‰ to −19.4 ± 2.3‰ during daylight and from −14.2 ± 1.64‰ to −20.1 ± 3.81‰ under twilight conditions. The Keeling plot intercept, representing the δ^13^C of the CO_2_ source, changed significantly under daylight (from −23.5‰ to −26.6‰; ANCOVA, *p* < 0.001, shift = 3.1‰) but not under twilight (from −22.6‰ to −25.5‰; ANCOVA, *p* = 0.178; shift = 2.98‰).

In contrast, CH_4_ concentration ranges from 2.05 ± 0.02 ppm to 2.06 ± 0.02 ppm under illumination and from 2.05 ± 0.03 ppm to 2.06 ± 0.03 ppm under twilight. The mean δ^13^CH_4_ values shifted by −2.21‰ towards lighter signatures from −54.74 ± 2.05‰ to −56.95 ± 2.60‰ during illumination and shifted by +1.3‰ towards heavier signatures from −50.2 ± 2.46‰ to −48.9 ± 4.01‰ under twilight conditions. Keeling plot analysis revealed that δ^13^C of the CH_4_ source, changed from 28.7‰ to 26.1‰ under illumination and from 3.3‰ to −29.4‰ under twilight but with a very weak linear relationship (R^2^ = 0.19–0.12, *p* < 0.05) under illumination and twilight conditions (R^2^ = 0.01–0.001, *p* < 0.05 before and *p* = 0.623 after incubation, respectively) with extremely wide confidence intervals for the intercept (>100‰) (Figure S1A–D). The dataset used for Keeling plot construction included measurements across different water content levels; a separate Keeling plot for each water content level also gave low R^2^ values (<0.19) and wide intercept confidence intervals (>100‰), so we analyzed the effect of water content on the δ^13^C of CO_2_ and CH_4_ in the gas samples during the experiments.

**Figure 6:**
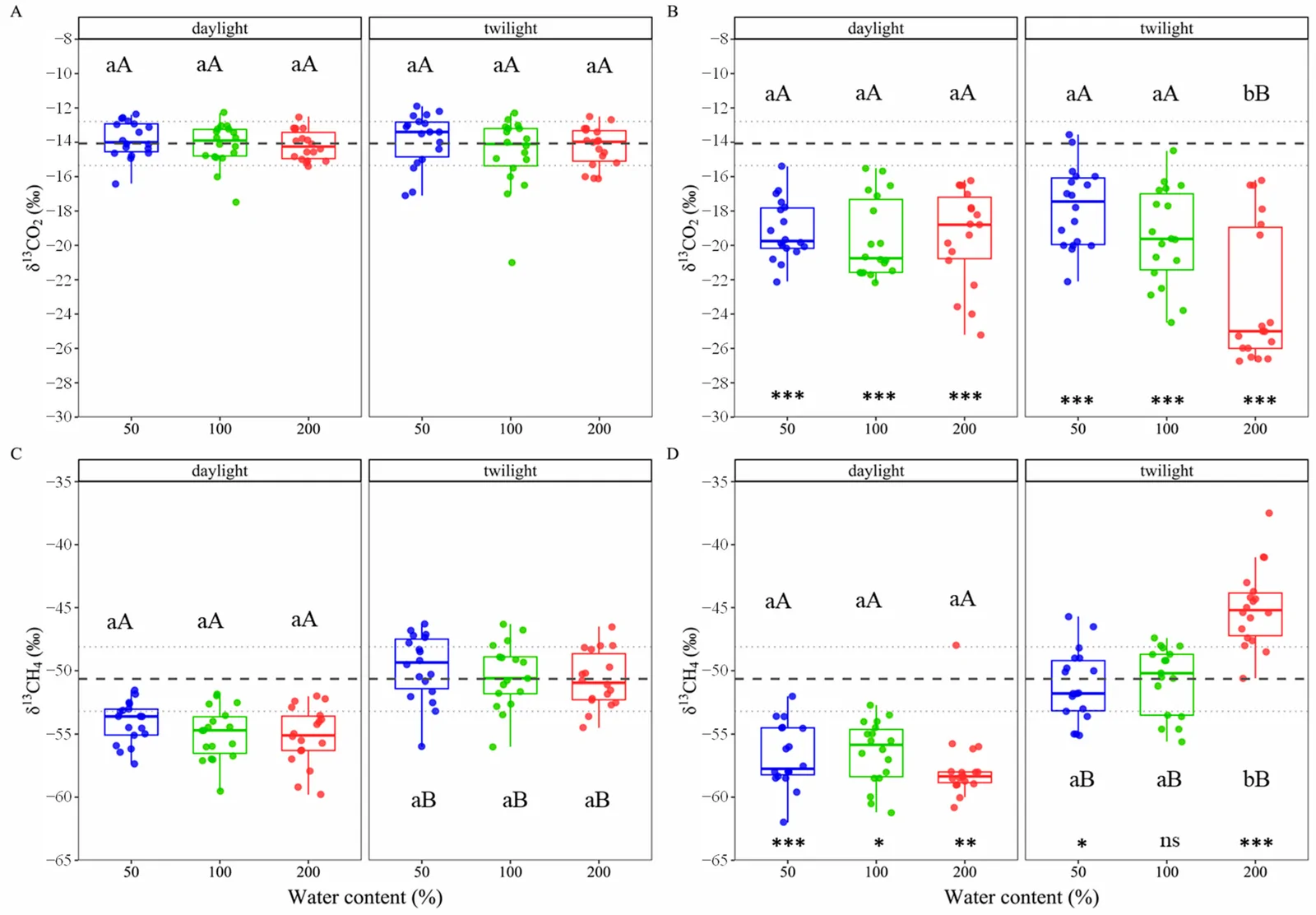
Boxplots of δ^13^CO_2_ and δ^13^CH_4_ from epiphytic lichens under daylight and twilight conditions at three thallus water content levels (50%, 100%, and 200% of dry mass) before (panels (**A**,**C**)) and after (panels (**B**,**D**)) 2-h incubation. Boxes represent the interquartile range (IQR, 25th–75th percentiles), horizontal lines indicate medians, whiskers extend to 1.5 × IQR, and individual points show raw observations (*n* = 54 per panel). Dashed horizontal lines indicate background (laboratory air) mean values (δ^13^CO_2_ and δ^13^CH_4_ in CO_2_ and CH_4_ in blank vials); dotted horizontal lines indicate background (laboratory air) mean ± standard deviation. Different lowercase letters indicate significant differences among water content levels within each light phase; different uppercase letters indicate significant differences between light phases within each water content level. Asterisks indicate significant differences in δ^13^C between before and after 2-h incubation for each treatment at the same lichen water content level: *** *p* < 0.001, ** *p* < 0.01, * *p* < 0.05, ns = not significant.

The boxplots in Figure 6A,C represent the carbon isotopic composition in CO_2_ and CH_4_ before 2 h of incubation. These samples were taken from the headspace of lichen-filled vials immediately after sealing and represent a mixture of the background isotopic composition of laboratory air and gases that may have been released within the first few minutes of incubation. Mean values of CH_4_ = 2.06 ± 0.02 ppm, δ^13^C-CH_4_ = −50.65 ± 2.54‰, CO_2_ = 553.97 ± 56.58 ppm, and δ^13^C-CO_2_ = −14.07 ± 1.28‰ in laboratory air vials (blanks, *n* = 29) were in agreement with atmospheric ambient levels.

Before incubation, no significant differences in δ^13^C-CO_2_ were detected between illumination and twilight conditions at any water content level (Wilcoxon test, 50%: *p* = 0.788; 100%: *p* = 0.646; 200%: *p* = 0.899). Furthermore, no significant effect of water content on δ^13^C-CO_2_ was observed for either condition (Dunn test with Bonferroni correction, all adjusted *p* = 1.000). Median δ^13^C-CO_2_ values ranged from −14.25‰ to −13.40‰ across all groups, confirming that the gas in the vials with the lichen inside was isotopically homogeneous before the incubation experiment.

To reveal the effect of lichen water content on δ^13^C-CO_2_ after daylight and twilight incubations, the Kruskal-Wallis test with pairwise comparisons (Dunn test, Bonferroni-corrected) was performed. Only under twilight conditions, signatures in gas samples after incubation with 200% lichen water content were significantly less negative (i.e., isotopically heavier) compared to 50% and 100% lichen water content and also heavier than in samples taken during daylight incubation at the same humidity of 200% (*p* < 0.05) (Figure 6B).

Before incubation, the δ^13^C-CH_4_ values differed significantly between the illumination and twilight experiments: headspace CH_4_ was more depleted in ^13^C under the illumination phase, whereas under the twilight phase it was close to background signatures (*p* < 0.001; Figure 6C). This difference likely reflects natural variability in the laboratory air between the two experimental runs, which were separated by a one-month interval.

After 2 h of incubation, under daylight conditions, lichen water content did not significantly affect δ^13^C-CH_4_ (two-way ANOVA, *p* = 0.18) (Figure 6D). However, under twilight conditions, a strong effect of water content was observed (*p* < 0.001), with a significant interaction between water content and incubation phase (daylight or twilight) (*p* < 0.001). Post-hoc analysis (Tukey HSD) revealed that samples with 200% water content had significantly higher δ^13^C-CH_4_ values compared to both variants of water content of 50% (Δ = +6.2‰, *p* < 0.001) and 100% (Δ = +5.7‰, *p* < 0.001). All samples incubated under twilight conditions showed significantly heavier δ^13^CH_4_ values compared to daylight-incubated samples at the same water content levels (*p* < 0.001).

Paired *t*-tests (for δ^13^C-CH_4_) and Wilcoxon tests (for δ^13^C-CO_2_) revealed different isotopic responses before and after incubation. δ^13^C-CO_2_ became significantly more negative (depleted) under all water contents (*p* < 0.001), with median shifts ranging from −4.05‰ to −11.03‰. δ^13^C-CH_4_ showed consistent depletion under daylight (−1.46‰ to −2.66‰, *p* < 0.05) and under twilight at 50% water content (−1.42‰, *p* < 0.05), no change at twilight at 100% (*p* = 0.6695), but strong enrichment at twilight at 200% (+5.70‰, *p* < 0.001). The largest CO_2_ depletion (−11.03‰) coincided with the largest δ^13^C-CH_4_ enrichment (+5.70‰) at twilight (200%), indicating coupled but opposing isotope effects under water-saturated, low-light conditions.

No significant differences in δ^13^C-CO_2_ and δ^13^C-CH_4_ were observed between the north- and east-exposed *Bryoria* thalli under any experimental conditions (Wilcoxon test, *p* ˃ 0.05 for all comparisons). Therefore, stem exposure was not considered a factor in analyses of isotopic data.

## 3. Discussion

### 3.1. Bryoria GHG Fluxes in the Field Conditions

This study assessed the climatic controls on GHG fluxes from *Bryoria* inhabiting larch trees in a permafrost-affected area over the growing season. We showed that precipitation (rain or fog) was the main climatic factor influencing GHG (CO_2_ and CH_4_) fluxes from the surface of lichen transplants (using *Bryoria simplicior* as an example) by altering the lichen thallus water content, which varied from 8% to 600% (r = 0.16) during the growing season of 2025. Due to the rare rain events and subsequent low thallus water content, the CO_2_ gas exchange of epiphytic lichen in field conditions has low variability (on average 0.4 mg C-CO_2_ m^−2^ h^−1^ per season, Figure 1). Esseen et al. [13] found that *Bryoria* retains up to 84% of its moisture as external capillary water between branches, which creates a buffer against rapid drying and allows photosynthesis to be maintained longer under low rainfall conditions. In addition to the complex branching structure of *Bryoria*, the extracellular polysaccharide matrix (ECM) of the lichen’s peripheral cortex, produced by lichen-associated bacteria on the surface of ascomycete hyphae, also contributes to the retention of significant volumes of external capillary moisture [12,13,40]. Air temperature showed no control on CO_2_ fluxes by *Bryoria* transplants (r = 0.05, Figure 3). According to Coxson & Coyle [17], *Bryoria* spp. is characterized by a relatively low temperature optimum for net photosynthesis (around +16 °C), reflecting its adaptation to cool climates. The authors also identified a critical physiological feature of *Bryoria* spp.—a decrease in the intensity of photosynthesis during prolonged (over 144 h) exposure to moisture saturation, which may limit the growth of these lichens in microniches with stagnant moisture [17].

Our results also showed that *Bryoria* consistently consumed methane during the season, with an average of −75 μg C-CH_4_ m^−2^ h^−1^. Regarding CH_4_ fluxes, neither air temperature (r = −0.02) nor precipitation (r = 0.1) altered the CH_4_ fluxes from the lichens. Stem exposure did not affect *Bryoria* GHG fluxes over the season (Figure 2).

### 3.2. GHG Fluxes of Bryoria in Laboratory Incubations and Hypothesis Regarding Methane Emission from the Lichen Surface

To assess potential GHG fluxes by *Bryoria* inhabiting larch under the modelled polar-day photoperiod conditions related to Tura (permafrost-affected Middle Siberia), we utilized the incubation method. The incubations showed the variation of CO_2_ flux rates from −19 to 140 μg C-CO_2_ g^−1^ h^−1^ and CH_4_ flux rates from −2.1 to 3.5 ng C-CH_4_ g^−1^ h^−1^.

#### 3.2.1. CO_2_ Fluxes

The emission of CO_2_ by *Bryoria* collected in the permafrost zone turned out to be 3.5 times lower (up to 140 µg C-CO_2_ g^−1^ h^−1^) compared to ELs of the non-permafrost zone (up to 511 µg C-CO_2_ g^−1^ h^−1^; [24]). Compared with European, Australian, and New Zealand epiphytic lichen species incubated in the dark at 21.5 ± 1.5 °C, *Bryoria* incubated under twilight conditions at +10 °C exhibited CO_2_ fluxes within the same range (e.g., 0.04–1.36 mg CO_2_ g^−1^ h^−1^) as those measured in species from Germany, Sweden, Australia, and New Zealand [20]. Moreover, CO_2_ fluxes from *Bryoria* were within the same range as CO_2_ fluxes (e.g., 24.7–307 µmol CO_2_ g^−1^ d^−1^ or 45.3–562.8 µg C-CO_2_ g^−1^ h^−1^) observed in tropical epiphytic species (*Leptogium*) in the lower montane rainforest in Panama [41].

In the incubation experiments involving gas sampling under illumination and twilight conditions, we illustrated the effects of stem exposure (the orientation of the stem section to which the lichen was attached) and varying thallus water contents. Under illumination, the east-exposed lichen with 200% thallus water content produced more CO_2_ compared to those assigned to the northern exposure (Figure 3A). Zotz et al. [42] showed that photosynthetic efficiency in *Sticta tomentosa* depends strongly on lichen exposure. This corresponds to the findings of Lange et al. [41], who reported the variations in lichen-derived CO_2_ fluxes from different stem exposures. The absence of net CO_2_ photoassimilation under illumination in our study, even at light levels above the compensation point, can be explained by the physiological characteristics of the lichen symbiosis [43]. First, the measured net CO_2_ flux represents the balance between photosynthetic CO_2_ uptake by the photobiont and respiratory CO_2_ release by the mycobiont. Under our experimental conditions, respiratory activity may have been sufficiently high to mask photosynthetic uptake, because the mycobiont’s biomass is over 90% and significantly greater than the photobiont’s (algae) biomass [2,44]. Second, the incubation temperature of +20 °C exceeds the photosynthetic temperature optimum for *Bryoria* (approximately +16 °C; [17]). This, combined with constant moisture availability (unlike field conditions), resulted in reduced photosynthetic efficiency (Figure 4A). Third, at high thallus water content (200%), the suprasaturation effect increases diffusive resistance to CO_2_, limiting net photosynthesis [41], but this effect was observed only under twilight conditions (Figure 4B). These three factors could therefore be at play in this context, alone or in combination.

The isotopic composition of CO_2_ helps identify the origin of observed flux data. The δ^13^C of CO_2_ became depleted under both light and twilight conditions. This 3‰ decrease after 2 h incubation indicates a substantial internal source of isotopically light carbon—most likely reflecting the predominance of catabolic processes, such as respiration with utilization of fungal reserves (mannitol, glycogen) characterized by the δ^13^C value of ca. −30‰ or lower (Figure 5A–D). The metabolism of these substantial pools, expressed as a proportion of the lichen’s dry mass, can markedly alter the isotopic composition of the CO_2_ accumulated within two hours. Fungus-specific polyhydric alcohols, such as mannitol and arabitol, which represent 2–10% of the thallus dry weight [45,46], are formed from ribitol, which is secreted in abundance by the algae, e.g., *Trebuxia* [9,47,48,49]. In the decreased fungal extracellular matrix (ECM), which is found in the medullary compartment of the lichen, fungal hyphae form tight wall-to-wall connections with photobiont cells, creating a concentration gradient that “pulls” fresh parts of ribitol out of the alga [50,51,52]. Fungal polyhydric alcohols provide both a substrate for the production of the fungal component and are oxidized during respiration to make ATP, which is important for growth and survival [50]. Arabitol is considered a more easily respired substrate than mannitol [53,54], with the latter recognized as a low-molecular-weight storage molecule [55]. Interestingly, *Trebouxia* rapidly ceases releasing ribitol into the environment and starts to gather sucrose for its own requirements when the partners (fungi and algae) are separated [56]. Hence, lichens exert control over the cell cycle of their photobionts, potentially regulating their growth, reproductive strategies, and metabolism [57,58]. In addition, polyols (e.g., mannitol and arabitol) prevent the formation of ice crystals inside hyphae at low temperatures, allowing lichens to survive in the Arctic and highlands (cryoprotection) [45,59,60]. Polyols also effectively block reactive oxygen species (ROS) [61]. Fungal polyols (mannitol) also help the fungus retain water and prevent damage to cellular structures when the lichen dries out completely (osmoprotection), which is critical for lichens that live in conditions of constant drying and wetting cycles [50,62,63]. In conjunction with fungal polyols, LEA-like proteins identified in *Trebouxia* [64] likely serve as molecular shields that, when paired with non-reducing sugars, substitute for water, maintaining the spatial separation between macromolecules [65], thereby reducing molecular congestion and preventing cellular collapse during dehydration. In our previous work, we partly confirmed the influence of lichen water content on CO_2_ fluxes [23]. Increasing thallus moisture leads to faster CO_2_ release and slower photoassimilation for lichens. In our recent study, 200%-hydrated thalli of *Evernia mesomorpha* stopped CO_2_ photoassimilation after two-hour daylight exposure, whereas a 200%-hydrated *Bryoria* collected from east-exposed branches demonstrated pure CO_2_ assimilation of up to −30 μg C-CO_2_ g^−1^ h^−1^ [23]. This difference in CO_2_ assimilation responses can result from the different photoperiod conditions in the previous study: 18-h day at full illumination at +18 °C and 6-h night in the dark at +14 °C [23]. Therefore, it might be possible that different photoperiod regimes and conditions may influence CO_2_ flux. In addition, the presence of carbon concentrating mechanisms (CCM) in *Trebouxia* [66] can slightly shift the ^13^C of ribitol (making it more enriched), since the alga more efficiently absorbs available carbon under conditions of limited gas diffusion (for example, with severe waterlogging of the thallus), which explains the range of ^13^C values of the CO_2_ source in the lichen in our study (Figure 6A). Using metatranscriptomic analysis, Cernava et al. [36] showed that the lichen-associated bacterial community can transition from a carbohydrate-based to a lipid-based substrate under stressful conditions (such as dehydration). Both algae and fungi can be the source of lipids (photobiont and mycobiont have different sets of lipids and, hence, different “storage facilities”). Thus, lipid metabolism in lichens can be compartmentalized amongst symbionts [67], and the ^13^CO_2_ signature is likely to alter depending on the lipid used.

#### 3.2.2. CH_4_ Fluxes

CH_4_ fluxes from the *Bryoria* surface were not significantly affected by any of the examined/studied parameters (lichen water content, stem exposure), except at 50% thallus moisture content, where north-exposed items emitted more CH_4_ than east-exposed items. The average CH_4_ emission rate after 2 h of illumination was approximately 0.6 ng C–CH_4_ g^−1^ h^−1^, with fluxes ranging from −0.5 to 2.1 ng C–CH_4_ g^−1^ h^−1^ (Figure 3A). These values were slightly lower than the CH_4_ emission rates from lichens growing on birch (*Betula pubescens* Ehrh.) in the non-permafrost zone (Krasnoyarsk). For example, *Evernia mesomorpha* exhibited CH_4_ emission rates of up to 4.3 ng C-CH_4_ g^−1^ h^−1^ [24]. In the current study, CH_4_ emission fluxes from *Bryoria* (growing on larch in the permafrost zone) incubated under twilight conditions at +10 °C were 2.7–467 times higher (up to 2.1 ng C-CH_4_ g^−1^ h^−1^) than those reported for lichen species collected in Germany, Sweden, Australia, and New Zealand (e.g., 0.01–1.75 ng CH_4_ g^−1^ h^−1^) and incubated in the dark at 21.5 ± 1.5 °C [20]. However, CH_4_ fluxes from *Bryoria* generally fell within the lower end of the range reported for terrestrial plants and lichens in other locations (0.3–370 ng CH_4_ g^−1^ h^−1^), including non-permafrost areas [23,26].

It has long been believed that CH_4_ production is carried out by methanogenic archaea in anoxic environments, primarily via acetoclastic, hydrogenotrophic, or methylotrophic pathways (such as in lake sediments) [68]. Recent studies have documented aerobic CH_4_ production in various organisms, including cyanobacteria and phytoplankton, suggesting that alternative light-dependent mechanisms may exist [21,29].

Considering that our experiments were carried out both under illumination and under twilight conditions, the key process, namely the change in the isotopic composition of methane (δ^13^C in CH_4_), and the observed effects are most likely associated with differences in the activity of photosynthesis, photorespiration of the photobiont, and other lichen partners functioning, including the lichen microbiome (Figure 4, Figure 5 and Figure 6). Despite the initial (before 2-h incubation) differences in the isotopic composition of the headspace CH_4_ under illumination and twilight conditions, clear differences in isotopic composition of CH_4_ were revealed at different treatments after 2-h incubation (Figure 6C,D). Butterbach-Bahl et al. [69] claim that the mechanism of CH_4_ emission from lichens under aerobic conditions seems to differ from the commonly known pathways of soil microbial formation under anoxic conditions. In contrast, Aschenbach et al. [38] consider methane release from lichen as a result of the decomposition of organic matter by microorganisms (methanogens) in anaerobic microfoci within the lichen. This, however, is unlikely to be attributed to *Bryoria* because it is growing in an oxygen-rich environment.

Furthermore, the results of the twilight and illumination experiments suggest that light conditions may influence the growth and methanotrophic activity of bacteria within the lichen microbiome. Nonetheless, it cannot be overlooked that this impact may be linked not to light per se, but to its specific components (for instance, the UV spectrum or other characteristics of light waves) (Figure 4). This claim has been supported by experimental investigations on pure cultures of *Methylosinus trichosporium*, which were significantly suppressed by illumination above 150 microeinsteins m^−2^ s^−1^ [70]. It is assumed that in light (intensity > 150 μmol m^−2^ s^−1^) methanotrophs do not oxidize methane, and CH_4_, initially produced by methanogens, accumulates in the system. When methanotrophic oxidation is suppressed, isotopically light methane (^12^CH_4_) accumulates, resulting in a more isotopically depleted methane signal. This is consistent with our data, in which mean δ^13^CH_4_ values shifted towards more depleted signatures (from −54.74 ± 2.05‰ to −56.95 ± 2.60‰) during the full-light phase (Figure 6C,D). This may point to the predominance of CH_4_ emission over oxidation during the 2-h incubation of *Bryoria*. During twilight (low illumination of 10 μmol m^−2^ s^−1^ and +10 °C), the inhibitory effect is alleviated or diminished, potentially enabling methanotrophs to oxidize isotopically light methane, thus influencing the overall isotopic composition. This is supported by a shift of the mean δ^13^CH_4_ readings towards heavier signatures from −50.2 ± 2.46‰ to −48.9 ± 4.01‰ under twilight conditions (Figure 6C,D). Interestingly, lichen water content has affected the isotopic composition of ^13^CH_4_ after 2-h incubation under twilight conditions (Figure 6D). Specifically, ^13^CH_4_ from the lichen at 50% water content becomes significantly more depleted (−51.1‰, *p* < 0.05) after 2 h incubation, whereas ^13^CH_4_ readings in the headspace of the flask with the lichen of 200% water content become more enriched (−45‰, *p* < 0.001).

Given our finding that CH_4_ emission is a light-associated phenomenon, it is likely to be correlated with the photosynthetic activity of the photobiont, which constitutes our primary hypothesis. According to Lenhart et al. [20], CH_4_ emissions can originate from photoautotrophic organisms in the cryptogamic covers such as bryophytes, cyanobacteria, and lichens, as has been shown in a series of microbial inhibitor experiments. The majority of *Bryoria* species are solely connected with members of the *Trebouxia simplex* group [71].

Besides the aforementioned photosynthesis-related CH_4_ emission in aerobic conditions, alternative mechanisms, such as microbial-mediated CH_4_ production, may also be relevant in the context of lichens.

In higher plants, an abiotic mechanism of CH_4_ generation under aerobic conditions via the reaction of methyl esters of pectin (–O–CH_3_) and hydroxyl radicals (•OH) has been described [26,71,72,73,74]. We propose that an analogous mechanism may operate in lichens. This hypothesis requires an accessible source of methylated substrates. Although the cell wall of *Trebouxia* (the lichen photobiont) is multilayered and contains cellulose, sporopollenin, specific polysaccharides, and proteins, it lacks pectin, amino sugars, and uronic acids [75]. However, González-Hourcade et al. [76] showed that *Trebouxia* secretes proteins into the extracellular matrix (ECM) that produce H_2_O_2_ and acidic polysaccharides containing uronic acids and sulfated sugars. Furthermore, Spribille et al. [77] noted that some acidic polysaccharides (including pectin-like compounds) and sulfated sugars are not detectable by conventional neutral sugar analysis but may be present in the lichen ECM, the composition of which remains poorly characterized. Therefore, the ECM—rather than the isolated algal cell wall—represents an accessible substrate for bacterial degradation [40] and a potential source of methylated molecules for subsequent abiotic CH_4_ emission.

Hydroxyl radicals (•OH), which attack methyl groups, are generated from reactive oxygen species (ROS). ROS are produced in mitochondria as a byproduct of metabolic activity, and stress accelerates this process [39]. In our experiments, CH_4_ emissions under combined light and +20 °C conditions were slightly higher than during the twilight phase (semi-dark, +10 °C) (Figure 4), suggesting that ROS production is more active under the combination of light and elevated temperature.

Fungi, including the lichen mycobiont, also generate ROS via enzyme systems such as AAO, NOX, and GMC oxidases [78]. The resulting H_2_O_2_ can form •OH through the Fenton reaction [22]. Importantly, this pathway can utilize methyl groups not only from pectin but also from proteins, lipids, and nucleic acids. According to Keppler et al. [72], the isotopic composition of methyl groups of plant pectin can range from −11 to −46‰. In our research, the isotopic composition of CH_4_ in the gas phase was enriched at 200% thallus water content (Figure 6D), but under twilight conditions the average δ^13^C-CH_4_ values shifted towards lighter signatures. Although the Keeling plot for CH_4_ was statistically unstable (Figure S1), the observed δ^13^C-CH_4_ values fall within the isotopic range of reported methyl groups. Therefore, this does not contradict the theory that the methyl groups of long-chain molecules found in the lichen EPS are the source of CH_4_ formation. Additionally, it has been shown that significant carbon emissions, including CH_4_ [22,79] and CO_2_ [80], are produced by fungal metabolic processes.

Thus, in the illumination experiment, CH_4_ fluxes represent a net value resulting from the combination of CH_4_ consumption by methanotrophs (which is light-inhibited by light due to ^12^CH_4_ oxidation) and the abiotic ROS-induced CH_4_ formation. Twilight conditions are favorable for CH_4_ production, as low illumination does not inhibit methanotrophic processes. Moreover, high lichen water content (200%) significantly improves the uptake of the lighter isotope ^13^CH_4_, as evidenced by a more enriched isotopic composition of CH_4_ (Figure 6D). We acknowledge that other methane emission/absorption processes may occur in lichens, as lichens are complex systems with a large number of participants that are often difficult or impossible to identify and have various functions within the lichen than free-living individuals.

A limitation of our flux calculations is the two-point linear approach, which does not allow a formal test of linearity. However, for CO_2_, the high Keeling plot R^2^ values (0.69–0.95) confirm the linearity. For CH_4_, low R^2^ (0.00–0.19) and wide confidence intervals for the intercept (>100‰) violate the assumption of two-component mixing [81]; this is consistent with the observed minimal changes in concentration (<0.5% of the background level) and the bidirectional nature of methane exchange within the lichen symbiosis. Therefore, we restrict CH_4_ interpretation to qualitative comparison of mean δ^13^C values. We also acknowledge that two-point field measurements do not directly identify leaks of pressure effects; however, all chambers were carefully sealed and used according to standard protocols, and any systematic errors were equally distributed across treatments, allowing robust relative comparisons. Future studies would benefit from high-frequency automated sampling to more accurately determine concentration dynamics and confirm linearity.

Thus, this study enhances the existing understanding of the aerobic mechanisms of CH_4_ production by some EL species (*Bryoria simplicior*) residing in the permafrost zone. Although seasonal measurements of GHG fluxes by *Bryoria* transplants showed a predominant emission of CO_2_ (0.4 mg C-CO_2_ m^−2^ h^−1^) and uptake of CH_4_ (−75 μg C-CH_4_ m^−2^ h^−1^), likely due to climatic conditions (low precipitation), incubation experiments showed that ELs mainly emit GHGs such as CO_2_ and CH_4_. In incubation experiments, CO_2_ emissions from *Bryoria* reached 140 µg C-CO_2_ g^−1^ h^−1^, while methane emissions reached 3.5 ng C-CH_4_ g^−1^ h^−1^. This discrepancy may be explained by differences in hydrothermal regimes: in field conditions, scarce precipitation and low air humidity result in low water content of lichens, limiting the activity of both methane producers and consumers, whereas in incubations, optimal emission conditions are established. Therefore, the incubation experiments reflect potential GHG fluxes under conditions that simulate a combination of specific photoperiod and lichen hydration levels. In this context, the direction of CO_2_ fluxes by ELs is critically reliant on the thallus water content and the duration of photoperiod, whereas CH_4_ fluxes are primarily light-dependent and show only a minor response to variations in the illumination status via the stem exposure or the hydration level of the thallus.

The above considerations point to the important role of microorganisms, yeasts, free-living algae, archaea, and fungi linked to the lichen, which in this context takes the form of a complex holobiont. These organisms contribute to the functioning of the lichen, such as facilitating gas exchange of GHGs by altering the substrate; for example, the bacterial community can switch from a carbohydrate substrate to a lipid substrate under stress conditions. In addition, organisms associated with the lichen host may contribute to carbohydrate metabolism (as observed in certain yeasts), enhance lichen stress resilience (through secondary metabolites generated by specific yeasts), and facilitate water retention, thereby maintaining lichen hydration, for example, by forming an extracellular bacterial polysaccharide matrix that may also stabilize microbial communities in cold and/or nutrient-poor environments. Our research demonstrated the challenge of functionally identifying the sources of CO_2_ and CH_4_ (algae?, fungi?, or bacteria?, etc.) inside the lichen as a multifaceted holobiont. Thus, in the context of understanding the gas exchange of GHGs in lichens (especially CO_2_ and CH_4_), characterization of the microbiome of lichen-associated bacterial and fungal taxa may assist in further functional studies in the context of epiphytic lichens, specifically *Bryoria*.

### 3.3. Comparison of Experimental Results Under Field and Laboratory Environments

Comparing the findings of both studies, it becomes evident that methane uptake predominated in natural environments over the daytime measurement period, both by *Bryoria* growing on larch bark (site D) and by *Bryoria* specimens transplanted onto larch trees at the Tura Experimental Station. In contrast, under artificial illumination, methane was consistently emitted throughout the entire incubation study. This phenomenon may be attributed to methane diffusing from permafrost-underlain soils and ascending along tree trunks, particularly those of larch, as proposed in the soil–tree–atmosphere continuum [27]. If so, methanotrophic communities associated with the *Bryoria* thallus would have a consistent source of methane under natural conditions and would actively metabolize it, as has been shown for bark-dwelling methanotrophs [82]. However, because bare-bark controls were not included, we cannot separate the contribution of *Bryoria* from that of the bark substrate.

In the incubation experiment, there is no such constant influx of methane, and therefore the methanotrophic community is less active. In addition, the hydration of the thallus under field conditions is predominantly lower (Figure 1) than in incubation experiments (Figure 4). Consequently, different conditions are established for the operation of methanotrophic and methanogenic microorganisms. Therefore, in incubation experiments, we examined the specific characteristics of CH_4_ gas exchange in *Bryoria* thalli at varying moisture levels. As previously found, highly hydrated thalli adversely affect CO_2_ photoassimilation; consequently, in incubation experiments conducted at various humidity levels of thalli, we also noted increased CO_2_ release. Thus, we believe that the findings of our experiments will provide a basis for larger-scale studies of CO_2_ and CH_4_ fluxes under changing hydrothermal conditions driven by global climate change.

## 4. Materials and Methods

### 4.1. Study Site and Sample Collection

**Figure 7:**
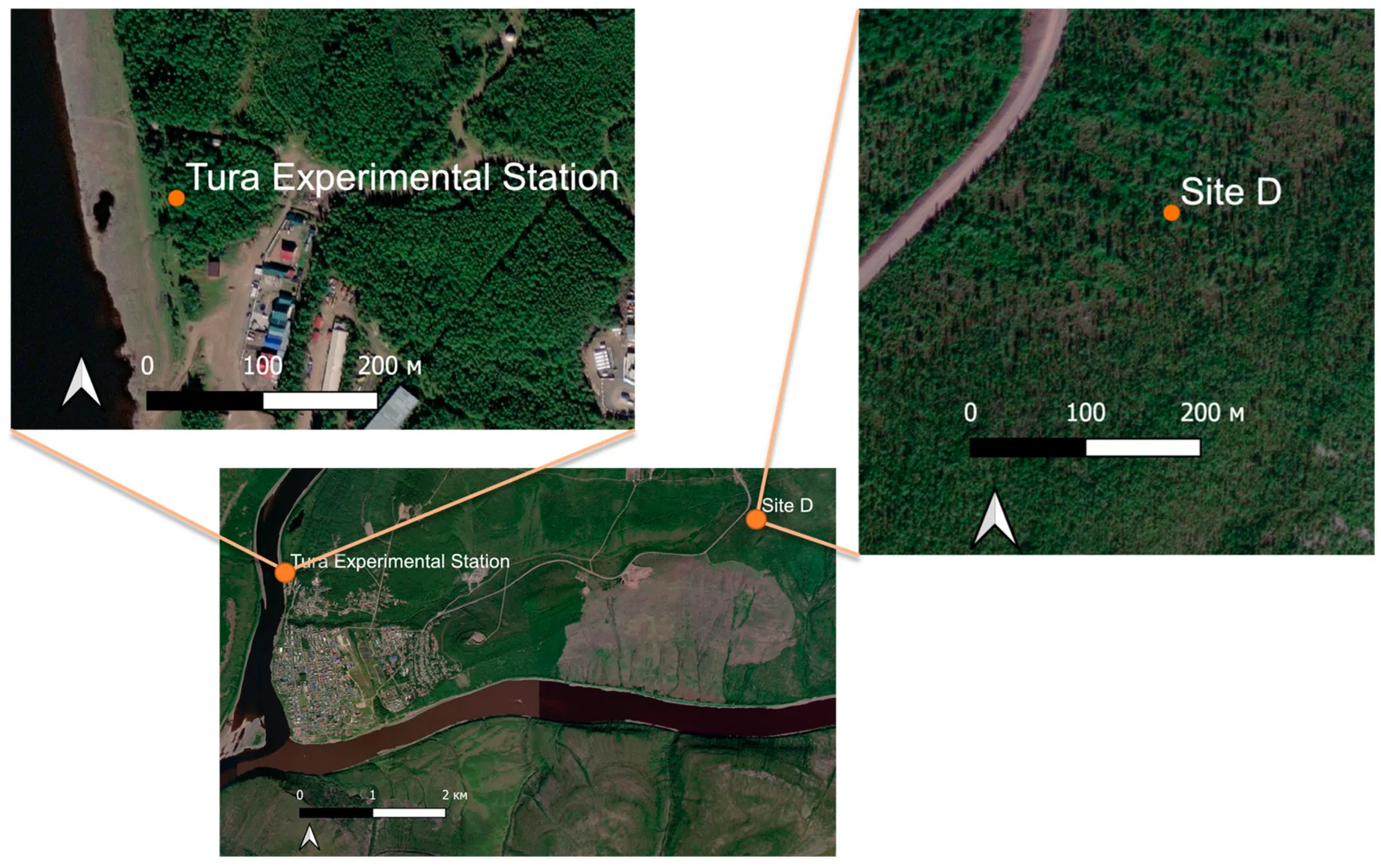
Map of site locations (Tura Experimental Station and site D) near Tura (64° N, 100° E; https://www.esri.com/, accessed on 14 May 2026).

The research was conducted in the continuous permafrost-affected area (Tura settlement, Krasnoyarsk region, Russian Federation) during the growing season 2025 (June 9–August 28) (Figure 7).

Study site D (64.294 N, 100.330 E, Figure 7) is located 8 km from the airport on the middle part of the slope (20°) of the northwest exposure. The tree stand is a pure larch forest. In the undergrowth, there are *Larix gmelinii* and *Pinus sibirica* Du Tour. In the underbrush, there are *Duschekia fruticosa* and *Rosa acicularis*. *Ledum palustre*, *Vaccinium uliginosum*, and *Vaccinium vitis-idaea* are dominant. In grass cover, there are *Lycopodium clavatum* L. and *Carex pediformis* var. macroura (Meinsh.) Kük. In moss cover, *Hylocomium splendens* (40%) dominates; *Pleurozium schreberi* (30%) co-dominates. The projective cover of lichens is 10%, including *Cladonia stellaris*, *Cladonia arbuscula*, *Cladonia rangiferina*, *Cladonia uncialis* (L.) F. H. Wigg., *Cetraria islandica* L. Ach., and *Flavocetraria cucullata* (Bellardi) Kärnefelt et A. Thell.

The geobotanical assessment of the studied area identified one of the most widespread EL species in permafrost area (*Bryoria simplicior*). The preliminary estimated projective cover of *Bryoria simplicior* on model trees ranged from 1 to 35% on stems and from 3 to 22% on branches [24]. ELs (*Bryoria*) on the bark were sampled at site D from several average larches, and composite samples for each stem exposure where the lichen was attached were formed and transported to Krasnoyarsk (Krasnoyarsk region, Russia) to conduct the incubation experiments on the potential GHG fluxes from ELs.

### 4.2. GHG Flux Measurements from Bryoria Transplants Under Field Conditions

**Figure 8:**
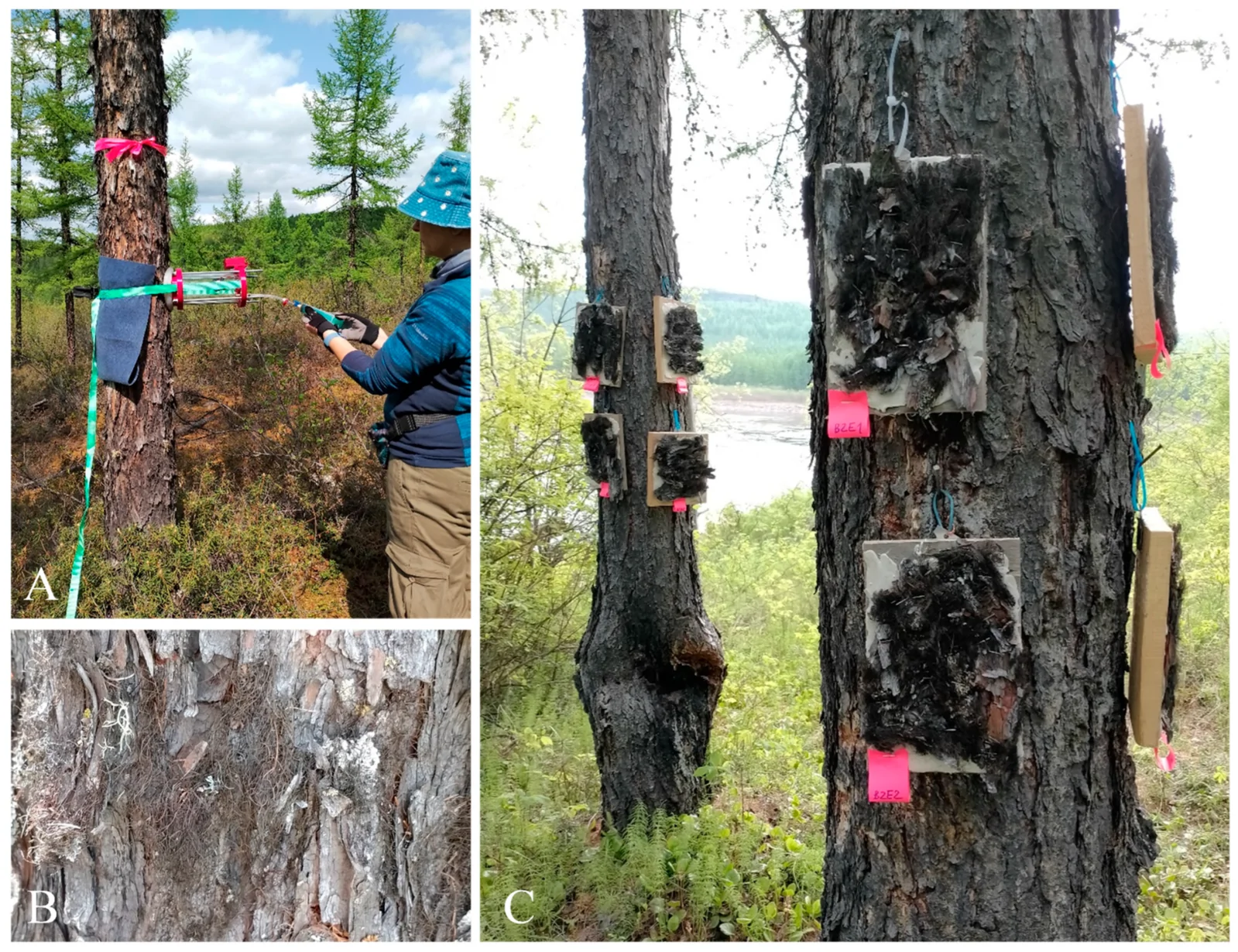
Actual GHG flux collection routine. (**A**), a collection of GHG fluxes from the surface of *Bryoria* inhabiting larch stems at site D (DOY 166); (**B**), *Bryoria* growing on the bark of larch (site D); and (**C**), *Bryoria* transplants affixed to the larch trees growing at Tura Experimental Station in order to conduct the seasonal series of transplant-based GHG flux measurements at the Tura Experimental Station from DOY 170 to 235.

From 9 June to 28 August 2025, seasonal measurements of GHG (CO_2_ and CH_4_) fluxes from *Bryoria simplicior* growing on the bark of larch trees in the larch forest located at site D (Tura) and from *Bryoria* transplants attached to the larch trees on the territory of the Tura Experimental Station were conducted in field conditions using an original transparent chamber for the collection of gas probes with Luer-Lock syringes (or gas-tight vials) (Figure 8). The time intervals for field measurements were 5–10 min. Immediately after collection, CO_2_, CH_4_, ^13^CO_2_, and ^13^CH_4_ were analyzed in gas probes using a Cavity Ringdown Spectrometer Picarro G2201-*i* (Picarro, Inc., Santa Clara, CA, USA). To conduct the incubation experiments and *Bryoria* transplantation experiment, at the remotely located study site D, as well as on the territory of the Tura Experimental Station (Tura settlement, Krasnoyarsk region), model larch trees were selected to collect ELs for incubation experiments and a *Bryoria* transplantation experiment. We transferred *Bryoria* thalli attached to north- and east-exposed larch bark fragments from the study site D to the Tura Experimental Station to create *Bryoria* transplants. *Bryoria* samples were found primarily on the northern and eastern sides of larch stems–possibly due to prevailing southern and western winds [23]. At Tura Experimental Station, *Bryoria* thalli on bark fragments were fixed on the chipboard planks using modeling clay and placed on the model larch trees with the diameters matching those of the model trees at site D [83]. The original orientation (exposure) of the lichen on the larch stems at site D was taken into account. The transplantation technique [84] facilitated maintaining environmental conditions similar to those at study site D and enhanced the frequency of measurements throughout the growing season. Transplantation to the Tura Experimental Station was necessary because regular access to site D was constrained by safety concerns and the lack of infrastructure for frequent sampling. Simultaneously with the GHG fluxes, the moisture content of the lichen thallus, PAR, and hydrothermal conditions were recorded.

GHG fluxes from *Bryoria* transplants were calculated per projected area of the lichen thallus inside the chamber using Formula (1).

(1) $$F=\frac{\left(C_{2}-C_{1}\right)\times V\times M}{24.5\times A\times t}*C,$$

where *F* is the flux rate of CH_4_ or CO_2_ (μg C-CH_4_ or C-CO_2_ m^−2^ EL area h^−1^), where the negative values correspond to CO_2_ or CH_4_ assimilation; *C_1_* is gas concentration (CH_4_ or CO_2_) before *t* of lichen exposure in the chamber (ppm); C_2_ is gas concentration (CH_4_ or CO_2_) accumulated during t of lichen exposure in the chamber (ppm); *V* is chamber volume (L); *M* is mole mass of CH_4_ or CO_2_ (16.043 or 44.01 g mole^−1^); 24.5 is a mole of air (L mole^−1^); *A* is lichen projected area in the chamber (m^2^); *t* is time of exposure of lichen in the chamber (h); *C* is a calculation per carbon for CO_2_ (12/44) or CH_4_ (12/16). The rates of GHG fluxes were calculated per projected area of the lichen for field measurements and per dry weight of EL for laboratory incubation conditions using a linear two-point approach, following standard static-chamber methodology [85,86], and should be considered as fluxes from the *Bryoria*-covered bark surface, thus representing a combined exchange of *Bryoria*, bark substrate, and bark-associated microorganisms.

### 4.3. Incubation Experiments

To ascertain the potential range of CO_2_ and CH_4_ fluxes in response to a simulated combination of polar-day photoperiod and temperature conditions, variable thallus water content, and differing orientation of the lichens on the stem (exposure), we employed the incubation method. The incubation approach enables the quantification of essential parameters in lichen C metabolism: the net rate of CO_2_ assimilation, determined by the balance of photosynthesis and photorespiration, and the rate of dark respiration. Assessing the dynamics of CO_2_ and CH_4_ concentrations, along with their stable isotopes (δ^13^C), offers a comprehensive indicator of lichen metabolic activity and their capacity to react to variations in environmental conditions such as humidity, temperature, and PAR [23,24,87,88,89].

*Sample preparation*. The cumulative samples of lichen *Bryoria* attached to the larch bark collected in Tura were carefully detached from the substrate and cleaned of particles. This yielded both cumulative *Bryoria* lichen samples and cumulative bark samples, which were intended to be incubated separately. Lichen samples attached to the bark of different stem exposures (northern and eastern) were divided, and composite lichen samples were formed for each stem exposure. After field collection, lichens were air-dried. To minimize osmotic stress, moistening was carried out by spraying with artificial rainwater [79,90]. The thalli were carefully separated from the substrate, cleaned of bark, moss, needles, etc., and formed composite samples. The composite samples were air-dried again and stored at room temperature. For the experiment, approximately 1 g (dry weight) aliquots of lichen material were weighed out in three biological replicates for each of the three thallus moisture levels (50%, 100%, and 200% of dry weight) and both stem exposures (northern and eastern). The calculated amount of artificial rainwater was added directly to 125 mL incubation vials (SIMAX 2070/M/100 mL, Kavalierglass, Inc., Sázava, Czechia). Before measurements, a 24-h preliminary aerobic incubation was conducted at room temperature to avoid a “resaturation” burst of GHG fluxes [91], to allow lichen metabolism to stabilize after wetting, and to reduce background variability in surface gas fluxes.

*Experimental conditions*. The samples were incubated under aerobic conditions (in slightly open vials) for three days in a programmable Peltier-cooled thermostat (Memmert, Schwabach, Germany). The daily polar day cycles were simulated using an integrated lighting system consisting of three LED lamps (Camelion LED 13-A60/830/E27, Camelion Batterien GmbH, Wildau, Germany) and one REPTILE UVB200 PT2341 lamp (Hagen Deutschland GmbH & Co. KG, Holm, Germany); the latter providing a high-intensity UV-B spectrum. The system provides the cumulative photosynthetically active radiation (PAR) of 100 μmol photons m^−2^ s^−1^ for 12 h in full illumination mode (day phase of photoperiod) at +20 °C and PAR of 10 μmol photons m^−2^ s^−1^ for 12 h at +10 °C in the mode of the minimum illumination period (twilight, night phase of photoperiod) [24].

Incubation conditions (two combined light-temperature phases as two photoperiod phases) were selected based on an analysis of hourly temperature and light conditions from the peak growing season months (June–July) recorded at a weather station in Tura (WMO ID: 24507 at http://aisori-m.meteo.ru/waisori/ accessed on 14 May 2026). In this study, the term “photoperiod” refers to the combined light-temperature regime used during incubation—specifically, a full-light phase (PAR 100 µmol·m^−2^·s^−1^, +20 °C) and a twilight phase (PAR 10 µmol·m^−2^·s^−1^, +10 °C)—designed to simulate the natural diurnal cycle of the polar day. The full-light phase duration (12 h) and the twilight phase duration (12 h) were assessed according to the threshold value of photosynthetically active radiation (PAR) of 50 µmol photons·m^−2^·s^−1^. For each day, the average day and night temperatures (20 °C and 10 °C) were calculated and used for incubation experiments. Analysis was performed in R (ver. 4.5.2) using the “dplyr” and “lubridate” packages.

*Gas sampling and analysis*. On each of the three days, the sample vials were removed from the incubator, weighed, and replenished with the required amount of artificial rainwater. They were then returned to the incubator for 2 h with their lids tightly sealed using rubber septa. Gas samples (22–25 mL) were collected from the headspace of each vial using Luer Lock syringes inserted through a septum. In the first experiment, gas samples were taken immediately before and 2 h after the incubation under the full-light phase at +20 °C; in the second experiment, gas samples were taken before and 2 h after the exposure under the low-light phase (10 µmol photons·m^−2^·s^−1^) at +10 °C. The concentrations of CO_2_ and CH_4_ and their isotopes δ^13^C (δ^13^CO_2_, δ^13^CH_4_) in gas samples were subsequently analysed using the Picarro G2201-*i* in the mode of simultaneous high-precision measurement of both gases and their isotopes (reproducibility: <0.16‰ for δ^13^CO_2_, and <1.15‰ for δ^13^CH_4_). The Picarro G2201-*i* analyzer automatically corrects for dilution and water vapor effects, providing dry air mole fractions and water-corrected δ^13^C values (as described in the G2201-*i* User Manual [92]). The rates of GHG fluxes were calculated per dry weight of EL using Formula (2).

(2) $$F=\frac{\left(C_{2}-C_{1}\right)\times V\times M}{24.5\times m\times t}*C,$$

where *F* is the flux rate of CH_4_ or CO_2_ (µg CH_4_ or CO_2_ g^−1^ dry weight EL h^−1^); *C_1_* is the concentration of gas (CH_4_ or CO_2_) before 2-h incubation of the lichens (ppm); *C_2_* is the concentration of gas (CH_4_ or CO_2_) after 2-h incubation of the lichens (ppm); *V* is the volume of the incubation vial (L); *M* is the molar mass of CH_4_ or CO_2_ (16.043 or 44.01 g mol^−1^); 24.5 is a mol of air (L mol^−1^); *m* is the dry weight of the EL in the incubation vial (g); *t* is the incubation time (2 h); *C* is a calculation per carbon for CO_2_ (12/44) or CH_4_ (12/16). The rate of GHG fluxes was expressed in µg C-CH_4_ or C-CO_2_ g^−1^ dry weight h^−1^. Since the difference in CO_2_ concentration is analyzed before and after 2-h incubation of EL in a closed vial, this is the result of various processes (heterotrophic respiration, photosynthesis, and photorespiration) [93,94]. Interpretation of the flow signs: for CO_2_, the positive *F* values correspond to pure CO_2_ release (dominance of respiratory processes). For CH_4_, positive *F* values correspond to emissions. Negative fluxes correspond to CO_2_ photoassimilation and methane consumption. Thus, the applied technique enables the quantification of the contribution of various physiological processes (photosynthesis, photorespiration, dark respiration, and methanotrophy) to the overall gas exchange of epiphytic lichens under controlled conditions. The isotopic source signature was estimated using the Keeling method [95], constructing a linear regression of δ^13^C against the inverse concentration (1/[C]). The regression was carried out in R with the ordinary least squares (OLS) approach (packages: stats, ggplot2, ggpubr). Confidence intervals for the intercept (the isotopic signature of the C source) were calculated at the 95% level using the confit() function. Normality and homogeneity of variances were tested using Shapiro–Wilk and Levene’s tests. For CO_2_ isotopic data, which were not normally distributed, non-parametric tests were applied (Kruskal–Wallis, Dunn’s post-hoc with Bonferroni correction, and Wilcoxon rank-sum test). For CH_4_ isotopic data, which met normality and homogeneity assumptions, parametric tests were used (two-way ANOVA with Tukey’s HSD post-hoc and paired *t*-tests).

## 5. Conclusions

The research provides the inaugural thorough characterization of the greenhouse gas emissions in *Bryoria simplicior* from the permafrost region.

This study demonstrates that precipitation translated into changes in EL thallus water content (varied from 8% to 600%), which was correlated with the GHG fluxes (weak but significant correlation) from the surface of the epiphytic lichen *Bryoria simplicior* in the permafrost zone of Middle Siberia. Under ambient conditions at the experimental station during the 2025 growing season, infrequent rain events led to consistently low thallus moisture, resulting in low variability in CO_2_ exchange (averaging 0.4 mg C-CO_2_ m^−2^ h^−1^) and a net uptake of methane (averaging −75 μg C-CH_4_ m^−2^ h^−1^). Stem exposure did not significantly influence these fluxes under field conditions.

Controlled incubation experiments designed to simulate the polar photoperiod revealed a wider range of potential metabolic activity. CO_2_ fluxes varied markedly, from net uptake to release (−19 to 140 μg C-CO_2_ g^−1^ h^−1^), and were critically different between combined light–temperature phases. Notably, CO_2_ emissions from the permafrost-zone *Bryoria* were 3.5 times lower than those previously reported for lichens in a non-permafrost zone. The direction of CO_2_ flux was linked to thallus moisture and combined light–temperature phase. At 50–100% humidity and the twilight phase, CO_2_ uptake (net assimilation) was observed. At 200% humidity (suprasaturation), only CO_2_ emission was recorded, which is associated with the suppression of photosynthesis due to high hydration level of lichen and/or temperature stress (+20 °C above the optimum). Under constant illumination (simulating a polar day), no CO_2_ photoassimilation was detected, likely due to the following reasons acting alone or in combination: 1. Respiratory activity of the mycobiont partner may have been sufficiently high to mask photosynthetic uptake; 2. The incubation temperature of +20 °C exceeds the photosynthetic temperature optimum for *Bryoria*; 3. At highly hydrated thalli, the suprasaturation effect increases diffusive resistance to CO_2_, limiting net photosynthesis. Isotope analysis (δ^13^C in CO_2_) indicated a powerful internal source of isotopically light carbon (down to –30‰ and below), presumably from fungal polyols (mannitol, arabitol)–reserve substances oxidized during respiration.

Methane fluxes from *Bryoria* were generally low and independent of thallus hydration or stem exposure, showing only a slight light-associated response. The observed emission rates (up to 3.5 ng C-CH_4_ g^−1^ h^−1^) were at the lower end of the global range reported for terrestrial plants and lichens and lower than those measured for lichen species in a non-permafrost region.

This research enhances our understanding of GHG dynamics in lichens of the permafrost zone. While *Bryoria* under ambient conditions at the experimental station showed net CH_4_ uptake during the study period, incubation experiments revealed its inherent capacity to both emit and consume CO_2_ and CH_4_. The direction and magnitude of CO_2_ fluxes are tightly linked to the interplay of thallus hydration and the combined light-temperature regime (photoperiod). Whereas CH_4_ fluxes showed a predominantly light-associated response.

In conclusion, *Bryoria*-associated samples showed measurable CO_2_ and CH_4_ exchange under field and controlled hydration/light–temperature conditions. These findings underscore the sensitivity of lichen-mediated GHG exchange to climatic variables and light regimes in permafrost ecosystems. However, further scaling and replication of the results are required to generalize these findings in the context of regional carbon budgets.

## Data Availability

Data are contained within the article or Supplementary Materials.

## Supplementary Materials

The following supporting information can be downloaded at https://www.mdpi.com/article/10.3390/plants15213328/s1, Figure S1: Keeling plots of δ^13^CH_4_ in a vial headspace of epiphytic lichens incubated in the daylight (panels A,B) and twilight conditions (panels C,D). Data are shown before (left column: A,C) and after (right column: B,D) the 2-h incubation. Dots are colored by thallus water content (50%—blue, 100%—green, 200%—red of dry weight) and have different shapes depending on stem exposure (north—circle, east—triangle) where the lichen was attached. Solid black lines are ordinary least squares (OLS) linear regression fits of δ^13^C to the inverse gas concentration (1/CO_2_). Regression equations and statistical parameters (R^2^, p) are shown.
