# Supplementary material for: CO2 and CH4 Fluxes of the Epiphytic Lichen Bryoria simplicior in a Permafrost-Affected Siberia: From Field Observations to Laboratory Incubations
Source: Plants (Basel). 2026 Oct 27;15(21):3328. doi: 10.3390/plants15213328 (PMC13765630; doi:10.3390/plants15213328)
Supplement: Supplementary file 1 [file plants-15-03328-s001.zip › plants-4476200-supplementary.pdf]

Supplementary Materials

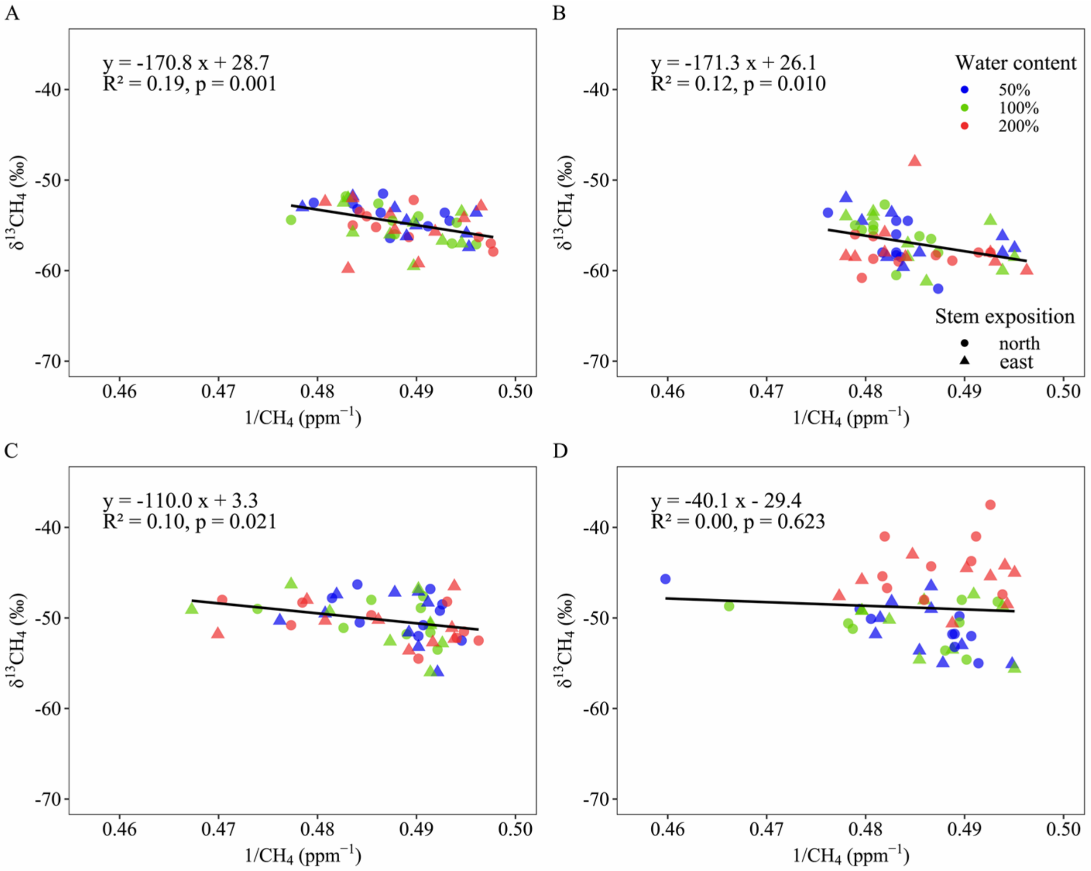

Figure S1. Keeling plots of $\delta^{13}CH_4$ in a vial headspace of epiphytic lichens incubated in the daylight (panels A–B) and twilight conditions (panels C–D). Data are shown before (left column: A, C) and after (right column: B, D) of the 2-h incubation. Dots are colored by thallus water content (50% – blue, 100% – green, 200% – red of dry weight) and have different shapes depending on stem exposure (north – circle, east – triangle) where the lichen was attached. Solid black lines are ordinary least squares (OLS) linear regression fits of $\delta^{13}C$ to the inverse gas concentration ($1/CO_2$). Regression equations and statistical parameters (R^2, p) are shown.
